# Supplementary material for: Coastal phytoplankton blooms expand and intensify in the 21st century
Source: Nature. 2023 Mar 1;615(7951):280–4. doi: 10.1038/s41586-023-05760-y (PMC9995273; doi:10.1038/s41586-023-05760-y)
Supplement: Supplementary file 1 — This file contains Supplementary Fig 1, and Supplementary Tables 1-3. [file 41586_2023_5760_MOESM1_ESM.pdf]

nature portfolio

https://doi.org/10.1038/s41586-023-05760-y

Supplementary information

Coastal phytoplankton blooms expand and intensify in the 21st century

In the format provided by the
authors and unedited

Supplementary Information for

Coastal phytoplankton blooms expand and intensify in the 21st century

Authors: Yanhui Dai[1,9], Shangbo Yang[1,9], Dan Zhao[1], Chuanmin Hu[2], Wang Xu[3], Donald M. Anderson[4], Yun Li[5], Xiao-Peng Song[6], Daniel G. Boyce[7], Luke Gibson[1], Chunmiao Zheng[1,8], Lian Feng[1,*]

Affiliations:

[1] School of Environmental Science and Engineering, Southern University of Science and Technology, Shenzhen, China

[2] College of Marine Science, University of South Florida, St. Petersburg, FL, USA.

[3] Shenzhen Ecological and Environmental Monitoring Center of Guangdong Province, Shenzhen, China

[4] Woods Hole Oceanographic Institution, Woods Hole, MA, USA

[5] School of Marine Science and Policy, College of Earth, Ocean, and Environment, University of Delaware, Lewes, DE, USA

[6] Department of Geographical Sciences, University of Maryland, College Park, MD, USA

[7] Bedford Institute of Oceanography, Fisheries and Oceans Canada, Dartmouth, Canada

[8] EIT Institute for Advanced Study, Ningbo, China

[9] These two authors contributed equally

*Correspondence to: fengl@sustech.edu.cn

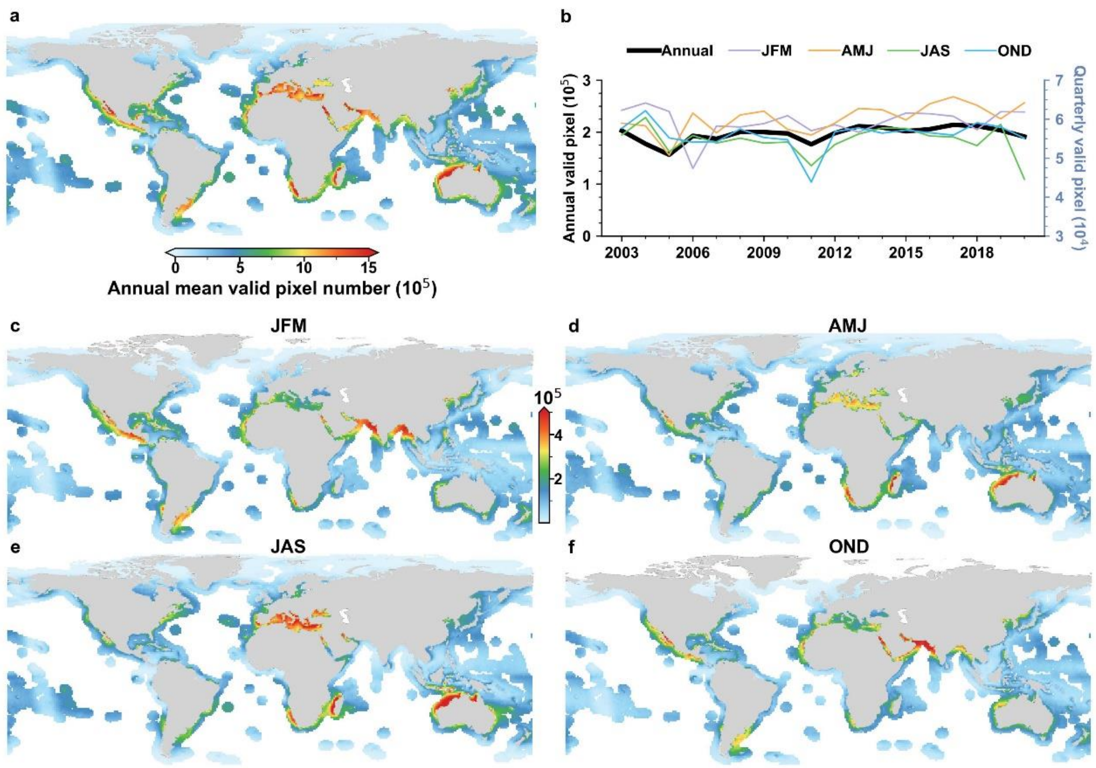

Supplementary Fig. 1 | The number of valid pixel observations (N_{vobs}) for global 1 °×1 °grid cells. (**a**) Global pattern of mean annual N_{vobs}, and the mean values for four quarters are shown from **c-f** (JFM: January, February, and March, AMJ: April, May, and June, JAS: July, August, and September, OND: October, November, and December). The interannual patterns from 2003 and 2020 are demonstrated in (**b**).

Supplementary Table 1. Accuracy assessments of the CIE-fluorescence algorithm for global coastal phytoplankton blooms detection.

Consistency between the satellite-detected bloom and the *in situ* recorded harmful algal bloom events from the HAEDAT dataset.

Year	*In situ* reported bloom event periods			*In situ* reported bloom events		
	Number of valid MODIS images within the bloom event period	Number of MODIS images with bloom detected within the bloom event period	Detection rate	Number of events	Number of satellite-detected events	Detection rate
2003	4325	1678	38.8%	113	98	86.7%
2004	3598	1496	41.6%	148	123	83.1%
2005	4719	2343	49.7%	137	107	78.1%
2006	3651	1596	43.7%	115	86	74.8%
2007	3524	1762	50.0%	107	92	86.0%
2008	3601	1853	51.5%	153	123	80.4%
2009	4245	2109	49.7%	152	117	77.0%
2010	4955	2907	58.7%	173	141	81.5%
2011	3676	2120	57.7%	136	115	84.6%
2012	4885	2607	53.4%	194	164	84.5%
2013	3881	2041	52.6%	151	129	85.4%
2014	5500	2781	50.6%	230	189	82.2%
2015	3930	2047	52.1%	166	136	81.9%
2016	3713	2098	56.5%	130	103	79.2%
2017	2184	1267	58.0%	95	75	78.9%
2018	2014	1095	54.4%	128	91	71.1%
2019	1911	971	50.8%	124	77	62.1%
2020	1642	1023	62.3%	157	103	65.6%
Total	65954	33794	51.2%	2609	2069	79.3%

Confusion matrix calculated by comparing visually delineated bloom pixels and those classified by the algorithm.

		Reference data		
		Bloom	Non-bloom	User accuracy
Classification data	Bloom	14235	197	98.63%
	Non-bloom	1231	13952	91.89%
	Producer accuracy	92.04%	98.61%	

Supplementary Table 2. Statistics for coastal algal blooms and nutrient enrichment for different countries. The long-term mean/median values for bloom count, affected area (both mean affected area, and the mean proportion of bloom-affected areas to the EEZ area in a country), and bloom frequency between 2003 and 2020 are listed, and the change rates for annual mean affected area and bloom frequency are estimated. The correlation coefficients (r) between annual bloom frequency and three possible anthropogenic nutrient sources (nitrogen and phosphate fertilizers and aquaculture production) are estimated, and the change rates for the three possible anthropogenic nutrient sources are listed. Change rates (in %) were calculated as the ratio between linear slope and the corresponding long-term mean values. Significant (*P*<0.05) change rates and correlation coefficients are indicated by "*". Blank cells represent no data for the nutrient source.

Continent	Country	Statistics for coastal algal blooms						Changes in nutrient enrichment and correlations with bloom frequency					
		Median bloom count	Affected area			Bloom frequency		Nitrogen		Phosphorous		Fishery production	
			Mean proportion (%)	Mean (km^2)	Change rate (%)	Mean	Change rate (%)	r	Change rate (%)	r	Change rate (%)	r	Change rate (%)
Africa	Namibia	25.06	41.02	461027	0.19	171971.6	1.66	0.33	17.71 *	0.55 *	10.49 *	0.32	5.93 *
	Mauritania	18.17	67.39	174068.2	0.08	133960.9	0.18						
	Eritrea	13.83	22.57	61522.3	-0.46	96139.8	-0.7	-0.24	14.46 *	-0.17	-10.87		
	Western Sahara	11.5	35.08	204614.2	0.62	66426.5	2.3						
	Tunisia	6.28	5	21700.5	-1.14	650.8	-2.92	-0.39	1.30 *	0.02	1.44 *	-0.14	15.18 *
	Angola	5.94	35.66	201642.8	-0.63	6419.6	-7.06 *	-0.35	7.71 *	-0.24	5.97 *	-0.38	18.15 *
	Guinea-Bissau	5.78	25.45	45489.6	0.65	7116.3	-0.06					0.98	
	South Africa	5.39	7.67	518010.2	0.77	4564.8	1.51	0.15	0.19	0.07	3.01 *	-0.1	1.75 *
	Senegal	4.94	35.46	105042.9	0.16	24390.6	1.02	0.11	9.36 *	-0.03	6.67	0.13	20.17 *
	Morocco	4.06	11.29	93135.6	0.26	5871.5	5.13	-0.31	-2.25	0.26	1.56	-0.17	-3.07
	Gambia	3.83	61.41	29757.4	-0.83	39074.3	3.18	-0.02	-10.31 *	-0.10	-10.29 *	0.12	4.83
	Libya	3.28	0.02	217.7	-10.87	0.1	-24.25	0.15	-3.63 *	0.08	-1.04	0.44	-20.01 *

Federal Republic of Somalia	3	21.12	416289.5	-2.45 *	6612.2	-12.88 *						
Egypt	2.89	0.52	9784	-0.01	171	-2.5	-0.26	-0.75 *	-0.68 *	2.16 *	-0.36	8.01 *
Guinea	2.89	19.62	34214.2	1.58	6652.1	6.09	0.20	8.83 *	0.31	20.38 *	0.62 *	15.48 *
Mozambique	2.83	3.49	68303.4	-0.44	217.2	-4.46	-0.36	4.57	-0.39	2.12	0.11	-9.60 *
Ivory Coast	2.72	13.45	32616.4	0.67	748.4	3.46						
Djibouti	2.56	24.25	5654.4	-0.54	14044.2	-4.26						
Madagascar	2.5	3.51	94234.9	0.98	85.2	4.61	0.19	12.48 *	0.43	6.14 *	-0.12	5.04 *
Sudan	2.44	2.48	6441.1	-5.54 *	37.3	-27.5 *	-0.68	19.81 *	0.75 *	-10.65	-0.31	13.38 *
Sierra Leone	2.33	4.29	14125.4	1.06	737	4.19					0.22	6.85 *
Democratic Republic of the Congo	2.22	4.8	3260.8	-3.02	2121.9	-10.77						
Tanzania	2.17	1.24	6792.2	-4.95	67.3	6.49	-0.13	7.11 *	-0.03	9.64 *	-0.1	12.42 *
Kenya	2	7.15	26194.7	-4.04	291.8	-9.83	-0.2	4.29 *	-0.14	3.07 *	-0.18	12.58 *
Ghana	1.83	6	19409	0.73	222	1.31	0.06	12.69 *	-0.24	11.06 *	0.37	20.16 *
Republic of the Congo	1.83	18.16	16198.4	-0.34	5050.3	-2.4						
Gabon	1.72	10.9	59618.6	-0.36	1469	-5.49	-0.09	17.47 *	-0.06	19.42 *	0.12	-5.80 *
Algeria	1.61	21.92	59061.3	2.1	5719.6	5.44	0.21	4.62 *	0.46	4.95 *	0.66 *	9.81 *
Benin	1.39	0.04	160.8	-2.58	31.2	-17.22	0.38	20.87	0.37	21.80 *	-0.14	22.96 *
Equatorial Guinea	1.22	0.06	33742	-0.65	106.8	-12.04					-0.16	11.20 *
Seychelles	1.22	0.28	53594.1	2.19	8.4	7.16	0.32	15.24 *	-0.09	13.56 *	0.09	-16.76 *
Cameroon	1.17	1.98	5825.2	-1.69	329.4	-3.18	-0.03	4.30 *	0.08	0.27	-0.18	13.94 *

	Liberia	1.06	0.09	8714.8	6.69	17.2	13.18					-0.1	12.84 *
	Nigeria	1.06	0.29	6994	2.56	35	6.1	0.30	8.03 *	0.35	9.93 *	0.06	11.19 *
	Togo	1	0.05	1795.2	-6.58	662.6	-13.2	0.14	3.20	0.29	3.15	-0.11	9.5
South America	Uruguay	32.22	28.39	123777.2	-0.21	209980.5	1.19	0.02	2.93 *	-0.11	0.26	0.50 *	9.73 *
	Argentina	30.61	24.24	856829.3	-0.14	157300.2	1.74 *	-0.12	2.44	-0.22	1.55	0.57 *	4.42 *
	Peru	9	26.09	671367.6	0.97 *	63383.8	0.88	-0.11	2.04 *	-0.28	0.87	0.24	10.16 *
	Venezuela	7.61	10.8	131165.9	0.82	1567.4	-1.86	0.02	-0.84	-0.02	4.58 *	0.06	2.93 *
	Chile	4.67	6.13	1037170.8	0.87	2436.2	1.86	-0.43	-0.49	0.13	-0.03	0.39	4.23 *
	Brazil	4.5	3.03	424457	0.72	522	-1.19	-0.41	4.25 *	-0.42	2.41 *	-0.47	6.46 *
	Guyana	4.33	19.81	39159.8	0.4	1448.9	3.33	0.51 *	3.33	0.03	4.94	-0.28	-3.84
	Ecuador	4.17	11.97	180124.7	1.42 *	25220.5	3.34 *	0.32	3.78 *	0.28	3.31 *	0.37	10.11 *
	Suriname	4.06	32.05	64345.5	0.88	7713.1	3.85	0.19	0.92	0.06	-0.37	-0.51 *	-7.72 *
	Colombia	1.67	9.83	234657.7	2.52	4644	10.63 *	-0.71 *	-1.82	-0.65 *	-1.52	0.36	5.01 *
Europe	Belgium	28.22	47.45	6746.9	-0.01	112504.1	1.21	0.12	1.37 *	-0.29	-7.20 *	0.09	-14.35 *
	Lithuania	20.94	39.91	6389	-0.01	96636.6	0.95	0.05	0.93 *	-0.03	1.96 *	0.26	3.98 *
	Estonia	20.28	27.12	25690.7	-0.03	39463.2	0.66	0.10	2.30 *	0.14	0.22	0.16	4.55 *
	Poland	20.22	22.17	23225.8	0.02	88075.8	0.81	0.23	1.61 *	0.05	-0.04	0.31	0.77
	Latvia	19.72	38.92	29864.4	0	94852.8	1.21	0.23	4.21 *	0.19	3.79 *	0.38	2.68 *
	Sweden	19.5	13.05	113196.6	0.05	56725	0.19	0.45	1.03 *	0.52 *	-0.69	-0.08	6.30 *
	Finland	18.06	17	61527.1	0.11	74326.5	0.46	0.11	-2.05 *	0.01	-4.35 *	0.77 *	0.73
	Germany	14.22	20.53	49258.7	-0.03	32960	0.07	0.20	-1.06 *	0.14	-1.06	-0.27	-4.51 *
	Romania	11.56	55.54	14129.6	-0.16	68700.4	-1.49	-0.20	3.68 *	-0.22	3.99 *	0.03	2.49 *
	Ukraine	11.5	24.33	73361.3	0.65	4193.7	1.23	-0.01	8.29 *	0	8.82 *	0.01	-1.69 *
	Netherlands	7.83	0.24	55202.1	0.01	34043.6	0.49	-0.09	-1.60 *	-0.14	-10.84 *	0.02	-1.31
	United Kingdom	7.44	0.99	1081078.2	-0.15	33177.8	-0.84	0.16	-0.57	0.01	-2.73 *	-0.09	1.11 *

	Norway	5.89	13.49	814601.1	0.27 *	14878.4	2.85 *	0.01	0.53	-0.35	-2.10 *	0.43	5.68 *
	Bulgaria	4.94	39.71	22562.5	0.76	4108	-0.46	-0.20	2.77 *	0.11	-2.42	0.01	11.68 *
	France	4.94	0.12	400405.8	0.66	1120.1	-8.54	0.09	-0.19	0.26	-2.91	0.15	-2.70 *
	Iceland	4.78	52.93	652099.9	0.15	18151.3	0.6	0.01	0.62	0.56 *	-1.35	-0.32	7.70 *
	Ireland	4.22	60.74	381835.4	0.24	22465.5	-1.3	0.56 *	-0.42	0.02	3.70 *	0.31	-3.71 *
	Monaco	3.83	8.06	11296.6	-1.58	17773	-4.9						
	Denmark	3.78	10.12	1308158.4	0.54	1326.7	1.06	0.10	1.03	0.11	-1.12	-0.16	-0.41
	Russia	3.78	4.66	3827546.5	0.92 *	1048.4	4.14	0.29	3.93 *	0.33	3.80 *	0.58 *	4.47 *
	Spain	3.17	7.71	348813.8	-0.1	5594.2	-2.76	-0.35	0.64	-0.45	-1.07	-0.28	1.06
	Croatia	2.72	1.7	5926.1	1.32	99.9	11.81	-0.11	-4.43 *	-0.32	-5.92 *	0.36	2.24 *
	Portugal	2.5	3.61	139547	-1.39	932.6	-3.52	-0.08	2.71 *	-0.44	-0.58	-0.29	3.83 *
	Italy	2.28	5.01	73782.7	-0.51	55.3	-2.92	-0.38	-1.61 *	-0.21	-3.69 *	-0.31	-0.96
	Greece	1.83	0.71	10673.8	0.21	4.2	2.37	0.30	0.06	-0.04	-4.53 *	-0.01	1.11 *
	Slovenia	1.44	27.57	2488.8	0.48	2431.1	3.96		-1.95 *		-4.87 *		1.89
	Albania	1.22	0.03	79	4.92	1.6	2.55	0.15	0.13	0.14	0.14	0	8.99 *
North America	Nicaragua	15.06	9.36	61579.2	0.16	4989.1	1.16	0.33	5.02 *	-0.12	3.62	0.21	7.94 *
	Mexico	8.5	10.54	732253	-1.01	3977.1	-2.65	-0.09	3.14 *	-0.10	4.80 *	-0.32	5.35 *
	El Salvador	6.33	53.1	73529.3	-0.76	53236.3	-1.87	0.37	-1.73	0.20	-0.72	-0.03	8.96 *
	Trinidad and Tobago	5.72	23.54	34288.2	-0.53	8884.6	-2.77	-0.20	2.91 *	-0.12	2.71	-0.06	-6.04
	Costa Rica	5.28	12.47	121516.7	-0.55	1634.2	3.67	0.21	1.24	-0.27	-4.30 *	0.02	-0.16
	Panama	4.11	20.45	99478.6	0.55	4232.4	0.82	-0.25	-2.11	-0.15	8.31 *	0.05	2.40 *
	Canada	3.78	8.63	2168838.9	0.35	340.3	-1.15	-0.12	3.65 *	0.03	4.14 *	-0.03	1.46 *
	United States	3.61	1.61	3368645.5	0.4	8078.8	2.57 *	0.11	0.72 *	0.13	0.39	-0.11	-1.95 *
	Dominican	3.56	0.13	1127.5	-2.3	53.3	-10.73	-0.25	4.78 *	-0.30	6.46 *	-0.38	4.12 *

	Republic												
	Guatemala	2.67	27.08	79267.8	-2.2 *	21563.4	-4.34	-0.09	3.73 *	-0.08	1.76	-0.10	6.96 *
	Belize	2.17	2.35	1519	5.13	294.5	11.57	-0.11	5.75	0.26	9.77 *	-0.13	-9.76 *
	Grenada	2.11	16.77	5910.7	-0.19	1423.8	-2.71					0.72	33.63
	Honduras	2.11	1.59	5622.2	5.3	55.1	21.83	0.12	5.38 *	0.38	7.06 *	0.23	4.07 *
	Cuba	1.78	0.57	5494.8	-0.27	5.2	-3.99	-0.48 *	4.52 *	-0.21	1.15	0.18	0.36
	Haiti	1.5	0.01	60.9	0.83	0.6	1.66					0.12	18.41 *
	Jamaica	1.22	0	239.1	-17.23	0.3	-23.86	0.59 *	-4.24 *	0.51 *	-3.30	0.16	-12.04 *
	Bahamas	1	0	2316.1	-2.27	0.2	-14.19	-0.39	20.90	0.45	3.90	0.12	-17.78 *
Asia	Iran	12.33	12.17	113102.6	-0.37 *	50174.6	-1.26	0.35	-5.01 *	0.24	-9.90 *	-0.14	9.98 *
	Pakistan	8.83	56.35	184064	0.01	84831.2	-4.57 *	-0.61 *	1.61 *	-0.28	3.37 *	-0.76 *	4.09 *
	United Arab Emirates	8	30.91	48825.2	-0.17	58710.8	0.12	-0.26	1.88	0.09	6.04	0.35	9.3
	Oman	7.72	35.58	433664.5	-0.02	65491.6	-2.07	-0.21	0.50	-0.34	3.47 *	0.50	-1.6
	Qatar	6.06	40.51	23533.9	-0.24	37929.5	-0.9	0.11	1.78 *	0.45	3.92	0.15	-4.11
	Bangladesh	4.72	14.08	34808	-0.08	8191.1	-1.93	-0.39	1.81 *	-0.02	8.51 *	-0.26	7.89 *
	Yemen	4.67	31.21	408632.2	-0.52	32021.6	-2.67	-0.42	0.22	-0.55	3.22	0.57	-17.91 *
	Myanmar	4.06	9.92	98652.9	0.96	1710.3	-1.6	-0.19	9.92 *	-0.05	11.52 *	-0.15	6.70 *
	India	3.72	8.22	544920.3	1.18	839.5	4.72	0.22	2.59 *	-0.02	2.31 *	0.04	6.32 *
	China	3.61	9.48	572574.2	0.78 *	6377	4.97 *	0.71 *	0.60 *	0.56 *	0.51	0.69 *	4.20 *
	Kuwait	3.5	25.84	3669.2	-2.2	5431.1	-6.92 *	-0.41	8.40 *	0.02	5.65	0.84 *	-4.40 *
	North Korea	3.44	20.53	71033.9	1.41	17632.6	5.45					0.23	1.47 *
	Bahrain	3.33	30.74	5740	-0.97	11541.5	-4.4 *	-0.18	0.27	0.34	-0.25	-0.08	9.35
	Japan	3.17	8.87	826334.6	1.63 *	5343.2	6.97 *	-0.45	-2.62 *	-0.45	-4.17 *	-0.31	-1.76 *
	Indonesia	3	11.53	1246061.3	0.33	503.2	5.02	-0.29	0.34	0.26	4.60 *	0.17	14.27 *
	Iraq	3	4.1	3821.8	-2.15	1943.7	-2.72		0.84		1.57		6.42 *

	South Korea	2.94	27.14	290054.3	1.15 *	12230.4	5.11 *	-0.38	-3.48 *	0.46	0.88	0.53 *	5.36 *
	Saudi Arabia	2.83	2.01	63445.6	-1.2 *	619.4	1.22	-0.40	-1.03	-0.33	-2.29 *	0.14	10.47 *
	Sri Lanka	2.61	21.77	160343.5	1.94	1112	7.88 *	-0.55 *	-3.15 *	-0.46	-1.75	0.40	14.47 *
	Vietnam	2.56	7.4	122546.8	1.51	730.7	9.73 *	0.29	0.74	-0.41	-1.04	0.61 *	7.49 *
	Malaysia	2.44	6.38	97565.2	-0.32	824.7	0.37	-0.27	-3.52 *	-0.16	-2.97 *	-0.2	4.96 *
	Thailand	2.39	4.83	42305.9	2.01	100.7	1.42	-0.04	0.87	-0.24	-1.05	0.47	-2.62 *
	Turkey	2.28	7.55	91400.9	0.62	4825.5	3.26	-0.08	2.56 *	-0.14	2.21 *	0.19	7.45 *
	Philippines	2.17	4.86	175019	-0.12	79.4	-2.37	0.55 *	0.93	0.15	0.97	-0.63 *	1.72 *
	Georgia	2	29.21	11638.9	1.66	4082.5	3.42	-0.38	1.71	-0.21	15.88 *	-0.17	16.50 *
	Brunei	1.94	6.59	8213.2	-2.6	1326.3	2.53	-0.12	-0.22	-0.01	2.38	-0.18	8.19 *
	East Timor	1.67	7.69	12643	-1.82	239.7	0.88						
	Cambodia	1.28	1.51	3126.2	-1.23	241.9	-8.33	-0.28	15.86 *	-0.46	4.45 *	-0.17	14.26 *
	Singapore	1.28	2.17	4467	0.86	1053.1	3						0.61
	Lebanon	1.14	0.01	37.5	-1.48	0.3	-3.12	0.05	1.31	0.71 *	5.01	-0.18	2.12 *
	Maldives	1.06	0.03	26005	-1.59	20.5	-4.28	-0.04	12.85 *	-0.15	1.88		
	New Zealand	2.5	0.83	1399329.3	0.4	1187.5	1.72	0.01	0.75	-0.01	-3.99 *	0.21	0.58
	Australia	2.28	2.52	1344721.1	1.48 *	237.4	7.54 *	0.05	1.66	-0.26	-2.53 *	0.28	5.48 *
Australia	Papua New Guinea	2	1.07	74129.4	0.81	5.6	1.28	0.18	5.26 *	-0.12	-0.42	0.16	16.43 *
	Fiji	1.97	0	2396.2	4.93	7.4	8.2	-0.29	-1.83	-0.40	17.59 *	-0.14	0.25
	Solomon Islands	1.22	0.05	12887.6	-0.96	2	-1.44					0.24	11.53 *
	Kiribati	1.11	0	4044.5	-5.21	0.1	-4.59					-0.04	-1.02

Supplementary Table 3. Statistics for coastal algal blooms for different large marine ecosystems (LMEs). The long-term median values for bloom count, affected area (both mean affected area, and the mean proportion of bloom-affected areas to the LME area), and bloom frequency between 2003 and 2020 are listed, and the change rates for annual mean affected area and bloom frequency are estimated. Significant (P<0.05) change rates are indicated by “*”.

Continent	LME	Statistics for coastal algal blooms in LMEs					
		Median bloom count	Affected area			Bloom frequency	
			Mean proportion (%)	Mean (km^2)	Change rate (%)	Mean	Change rate (%)
Africa	Benguela Current	15.67	24.51	843231.7	-0.05	30717.5	-0.81
	Canary Current	11.61	16.02	566227.7	0.28	29934.5	2.34
	Red Sea	7.78	5.83	118368.5	-1.01 *	92.1	-13.19 *
	Agulhas Current	4.33	4.13	403956.5	0.66	201	-2.11
	Somali Coastal Current	3.22	6.68	261710.6	-2.45	1769.6	-8.54
	Guinea Current	2.39	2.77	271950.1	0.37	167.1	1.83
South America	Patagonian Shelf	29.44	32.16	1017944.1	-0.03	165957.7	1.75 *
	Humboldt Current	5.89	17.29	1460295.6	0.87 *	14199.2	4.18 *
	South Brazil Shelf	5.83	10.69	195281.5	1.93 *	6751.1	1.77
	Caribbean Sea	4.94	4.01	245672.3	1.09 *	9.5	3.23
	North Brazil Shelf	4	10.31	418294.1	-0.08	3241.6	0.39
	East Brazil Shelf	2.56	1.06	28324.4	-1.53	42.4	-6.83
Europe	Baltic Sea	19.33	21.27	287420.2	0.05	59813.9	0.98
	North Sea	8.39	49.26	603787.1	0.03	37593	0.22
	Faroe Plateau	6.11	63.38	98619.6	-0.17	27790	-5.81 *
	Celtic-Biscay Shelf	6.06	35.2	626746.1	0.08	28370.1	-0.05
	Iceland Shelf and Sea	5.5	41.66	363696	0.01	17931	-0.97
	Iberian Coastal	3.89	30.76	226806.2	-0.35	19758.8	-0.41

	Black Sea	2.94	37.26	234015.4	0.93	4912.8	3.17
	Mediterranean Sea	2.67	6.85	366176.1	0.05	259	-0.19
North America	Northeast U.S. Continental Shelf	19.56	20.87	269082.8	0.01	121750	0.79
	Gulf of California	18.5	19.74	140395.8	-0.43	91034.8	-1.48
	Scotian Shelf	7.28	41.96	245237.6	0.04	42320.7	0.55
	Gulf of Alaska	6.39	18.72	853494.7	0.95 *	18128.9	1.61
	California Current	6.17	11.79	505938	-0.87	5360.5	-0.78
	Gulf of Mexico	5.78	15.62	287663.7	0.92	1242.2	-0.15
	Pacific Central-American Coastal	4.89	9.68	1010130.7	-0.25	13196.8	-0.36
	Southeast U.S. Continental Shelf	4.06	15.15	71364.4	-1.56	1744	-7.41
	Aleutian Islands	3.89	2.6	190848.3	0.77	16631.6	2.27
	Labrador - Newfoundland	3.72	19.2	633062.1	0.11	16548.4	2.03
	East Bering Sea	3.22	4.48	869397	0.61	13352.9	1.64
	Hudson Bay Complex	2.11	9.51	322939.1	0.23	156.4	0.79
	Insular Pacific-Hawaiian	1.08	0	79.5	8.67	0	9.74
Asia	Sea of Okhotsk	6.44	35.44	1336794.1	0.15	30905.5	0.96
	West Bering Sea	6.39	36.33	602295.4	0.43	25203.2	-0.29
	Arabian Sea	6.33	20	1794829	-0.08	27411.8	-3.25 *
	Oyashio Current	6.28	35.89	455862.6	0.51 *	40237.8	6.53 *
	Yellow Sea	3.78	45.89	301171.5	0.27	16881.8	2.35
	East China Sea	3.39	30.63	367395.8	0.78	7534.6	5.77 *
	Bay of Bengal	3.33	7.88	550704.2	1.47	421	3.08
	South China Sea	2.72	8.28	398792.5	0.91	216.1	3.1
	Sulu-Celebes Sea	2.67	8.87	136299.1	0.07	210.4	-1.36
	Gulf of Thailand	2.44	6.72	60262.5	0.95 *	116.3	0.25
	Kuroshio Current	2.39	5.61	254052.4	1.45 *	2910.4	3.37

	Indonesian Sea	2.33	14.06	532591.4	-0.19	613.8	3.85
	Sea of Japan	1.94	28.84	627238.8	2.41 *	7301	9.56 *
Australia	New Zealand Shelf	4.06	27.49	480335.9	-0.35	8227.1	-1.15
	North Australian Shelf	2.5	20.45	289141.3	0.1	1772.1	0.46
	West Central Australian Shelf	2.5	4.54	39420.2	6.67 *	27.9	21.62 *
	Southeast Australian Shelf	2.33	32.04	621942.3	1.15	5613.9	3.51
	Northwest Australian Shelf	2.22	5.8	120805.9	1.2	376.8	0.34
	East Central Australian Shelf	1.89	10.68	125204.8	1.26	905.2	1.95
	South West Australian Shelf	1.72	1.07	60504.6	10.09 *	33.3	28.76
	Northeast Australian Shelf	1.44	0.6	30317.1	2.08	31.7	2.31
