# Supplementary material for: Coastal phytoplankton blooms expand and intensify in the 21st century
Source: Nature. 2023 Mar 1;615(7951):280–4. doi: 10.1038/s41586-023-05760-y (PMC9995273; doi:10.1038/s41586-023-05760-y)
Supplement: Supplementary file 2 — Reporting Summary [file 41586_2023_5760_MOESM2_ESM.pdf]

nature portfolio

Corresponding author(s): Lian Feng
Last updated by author(s): Nov 23, 2022

Reporting Summary

Nature Portfolio wishes to improve the reproducibility of the work that we publish. This form provides structure for consistency and transparency in reporting. For further information on Nature Portfolio policies, see our Editorial Policies and the Editorial Policy Checklist.

Statistics

For all statistical analyses, confirm that the following items are present in the figure legend, table legend, main text, or Methods section.

n/a	Confirmed	
☐	☒	The exact sample size (*n*) for each experimental group/condition, given as a discrete number and unit of measurement
☐	☒	A statement on whether measurements were taken from distinct samples or whether the same sample was measured repeatedly
☐	☒	The statistical test(s) used AND whether they are one- or two-sided<br>*Only common tests should be described solely by name; describe more complex techniques in the Methods section.*
☒	☐	A description of all covariates tested
☒	☐	A description of any assumptions or corrections, such as tests of normality and adjustment for multiple comparisons
☐	☒	A full description of the statistical parameters including central tendency (e.g. means) or other basic estimates (e.g. regression coefficient) AND variation (e.g. standard deviation) or associated estimates of uncertainty (e.g. confidence intervals)
☐	☒	For null hypothesis testing, the test statistic (e.g. *F*, *t*, *r*) with confidence intervals, effect sizes, degrees of freedom and *P* value noted<br>*Give P values as exact values whenever suitable.*
☒	☐	For Bayesian analysis, information on the choice of priors and Markov chain Monte Carlo settings
☐	☒	For hierarchical and complex designs, identification of the appropriate level for tests and full reporting of outcomes
☒	☐	Estimates of effect sizes (e.g. Cohen's *d*, Pearson's *r*), indicating how they were calculated

Our web collection on statistics for biologists contains articles on many of the points above.

Software and code

Policy information about availability of computer code

Data collection	The satellite data were obtained from the U.S. National Aeronautics and Space Administration (NASA) Goddard Space Flight Center (GSFC).
Data analysis	SeaDAS (Version 7.5) were used to analyze the satellite images.

For manuscripts utilizing custom algorithms or software that are central to the research but not yet described in published literature, software must be made available to editors and reviewers. We strongly encourage code deposition in a community repository (e.g. GitHub). See the Nature Portfolio guidelines for submitting code & software for further information.

Data

Policy information about availability of data

All manuscripts must include a data availability statement. This statement should provide the following information, where applicable:

- Accession codes, unique identifiers, or web links for publicly available datasets
- A description of any restrictions on data availability
- For clinical datasets or third party data, please ensure that the statement adheres to our policy

The MODIS Aqua data can be obtained from the U.S. National Aeronautics and Space Administration (NASA) Goddard Space Flight Center (GSFC).
The in situ reported HAB data are available from events from http://haedat.iode.org.
The Exclusive economic zones (EEZs) dataset is available at https://www.marineregions.org/download_file.php?name=World_EEZ_v11_20191118.zip.
The boundaries of large marine ecosystems (LMEs) were obtained from https://www.sciencebase.gov/catalog/item/55c77722e4b08400b1fd8244.

Annual data between 2003 and 2019 on synthetic fertilizer use, including nitrogen and phosphorus, are available from https://ourworldindata.org/fertilizers.
Annual aquaculture production includes cultivated fish and crustaceans in marine and inland waters, and sea tanks, and the data between 2003 and 2018 are available from https://ourworldindata.org/grapher/aquaculture-farmed-fish-production.
The dataset is available from https://psl.noaa.gov/enso/mei/.

Human research participants

Policy information about studies involving human research participants and Sex and Gender in Research.

Reporting on sex and gender	*Use the terms sex (biological attribute) and gender (shaped by social and cultural circumstances) carefully in order to avoid confusing both terms. Indicate if findings apply to only one sex or gender; describe whether sex and gender were considered in study design whether sex and/or gender was determined based on self-reporting or assigned and methods used. Provide in the source data disaggregated sex and gender data where this information has been collected, and consent has been obtained for sharing of individual-level data; provide overall numbers in this Reporting Summary. Please state if this information has not been collected. Report sex- and gender-based analyses where performed, justify reasons for lack of sex- and gender-based analysis.*
Population characteristics	*Describe the covariate-relevant population characteristics of the human research participants (e.g. age, genotypic information, past and current diagnosis and treatment categories). If you filled out the behavioural & social sciences study design questions and have nothing to add here, write "See above."*
Recruitment	*Describe how participants were recruited. Outline any potential self-selection bias or other biases that may be present and how these are likely to impact results.*
Ethics oversight	*Identify the organization(s) that approved the study protocol.*

Note that full information on the approval of the study protocol must also be provided in the manuscript.

Field-specific reporting

Please select the one below that is the best fit for your research. If you are not sure, read the appropriate sections before making your selection.

☐ Life sciences ☐ Behavioural & social sciences ☒ Ecological, evolutionary & environmental sciences

For a reference copy of the document with all sections, see nature.com/documents/nr-reporting-summary-flat.pdf

Ecological, evolutionary & environmental sciences study design

All studies must disclose on these points even when the disclosure is negative.

Study description	This study developed a novel method to map global coastal algal blooms and used this tool to examine satellite images between 2003 and 2020, addressing three fundamental questions: 1) where and how frequently have global coastal oceans been affected by phytoplankton blooms? 2) have the blooms expanded or intensified over the past two decades, both globally and regionally? and 3) what are the potential drivers?
Research sample	Three separate samples were selected. 1) MODIS Aqua images were used to develop the phytoplankton bloom extraction algorithm, 2) MODIS Aqua images and were used to verify the reliability of the algorithm and the accuracy of the phytoplankton bloom extraction results, and 3) in situ reported HAB events from the HAEDAT dataset were used to validate the accuracy of the phytoplankton bloom extraction results.
Sampling strategy	A total of 115 MODIS Aqua images were selected from the different locations where coastal phytoplankton blooms have been recorded in the published literature, of which 80 were used for algorithm development and 35 were used for algorithm validation. A total number of 2609 HAB events that occurred in the coastal area were selected from the HAEDAT dataset.
Data collection	The HAEDAT dataset is a collection of records of harmful algal bloom (HAB) events , maintained under the UNESCO Intergovernmental Oceanographic Commission and with data archives since 1985.
Timing and spatial scale	The satellite data were acquired from different seasons and across various phytoplankton bloom magnitudes between 2003 and 2020, and HAB data from 2003 to 2020 in the HAEDAT dataset were used.
Data exclusions	No data were excluded from analysis.
Reproducibility	Our results could easily be reproduced with existing datasets.
Randomization	Excluding data affected by clouds, a total of 0.76 million MODIS Aqua images from 2003 to 2020 were used to extract phytoplankton blooms in global coastal area.
Blinding	Not applicable in our study.

March 2021

Did the study involve field work? ☐ Yes ☒ No

Reporting for specific materials, systems and methods

We require information from authors about some types of materials, experimental systems and methods used in many studies. Here, indicate whether each material, system or method listed is relevant to your study. If you are not sure if a list item applies to your research, read the appropriate section before selecting a response.

Materials & experimental systems

n/a	Involved in the study
☒	☐ Antibodies
☒	☐ Eukaryotic cell lines
☒	☐ Palaeontology and archaeology
☒	☐ Animals and other organisms
☒	☐ Clinical data
☒	☐ Dual use research of concern

Methods

n/a	Involved in the study
☒	☐ ChIP-seq
☒	☐ Flow cytometry
☒	☐ MRI-based neuroimaging
